# The Strange Case: The Unsymmetric Cisplatin-Based Pt(IV) Prodrug [Pt(CH_3_COO)Cl_2_(NH_3_)_2_(OH)] Exhibits Higher Cytotoxic Activity with respect to Its Symmetric Congeners due to Carrier-Mediated Cellular Uptake

**DOI:** 10.1155/2022/3698391

**Published:** 2022-12-30

**Authors:** Elisabetta Gabano, Ilaria Zanellato, Giulia Pinton, Laura Moro, Mauro Ravera, Domenico Osella

**Affiliations:** ^1^Dipartimento per lo Sviluppo Sostenibile e la Transizione Ecologica, Università del Piemonte Orientale, Piazza Sant'Eusebio 5, Vercelli 13100, Italy; ^2^Dipartimento di Scienze e Innovazione Tecnologica, Università del Piemonte Orientale, Viale Michel 11, Alessandria 15121, Italy; ^3^Dipartimento di Scienze del Farmaco, Università del Piemonte Orientale, Via Bovio 6, Novara 28100, Italy

## Abstract

The biological behavior of the axially unsymmetric antitumor prodrug (*OC*-6-44)-acetatodiamminedichloridohydroxidoplatinum(IV), **2**, was deeply investigated and compared with that of analogous symmetric Pt(IV) complexes, namely, dihydroxido **1** and diacetato **3**, which have a similar structure. The complexes were tested on a panel of human tumor cell lines. Complex **2** showed an anomalous higher cytotoxicity (similar to that of cisplatin) with respect to their analogues **1** and **3**. Their reduction potentials, reduction kinetics, lipophilicity, and membrane affinity are compared. Cellular uptake and DNA platination of Pt(IV) complexes were deeply investigated in the sensitive A2780 human ovarian cancer cell line and in the corresponding resistant A2780cisR subline. The unexpected activity of **2** appears to be related to its peculiar cellular accumulation and not to a different rate of reduction or a different efficacy in DNA platination and/or efficiency in apoptosis induction. Although the exact mechanism of cell uptake is not fully deciphered, a series of naïve experiments indicates an energy-dependent, carrier-mediated transport: the organic cation transporters (OCTs) are the likely proteins involved.

## 1. Introduction

**Figure 1:**
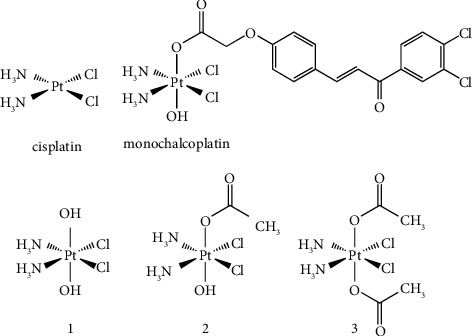
Chemical structures of the platinum complexes studied (cisplatin and **1**–**3**) and monochalcoplatin.

Today, systemic anticancer treatment is oriented towards targeted and immune therapy, with the aim of achieving precision medicine [1, 2]. However, classic chemotherapeutic agents, especially DNA-damaging ones, still play an important role in the treatment of more aggressive solid tumors, especially in combination therapy. Among these drugs, cisplatin (i.e., [PtCl_2_(NH_3_)_2_], (*SP*-4-2)-diamminedichloridoplatinum(II), Figure 1 and S1 Supplementary Material) and its counterparts are very active against many solid tumors but suffer severe drawbacks such as poor solubility and stability, lack of selectivity that causes severe side effects, and possible intrinsic or acquired chemoresistance [3, 4].

In recent decades, Pt(IV) derivatives have represented an interesting field of research. They have a low-spin d^6^ octahedral geometry and exhibit higher kinetic inertness, being less hydrolyzed or ligand substituted with respect to the Pt(II) parents. They reach almost intact cancer cells, where they are activated by 2e^−^ reductive elimination to release the corresponding Pt(II) cytotoxic agent and the two axial ligands, thus acting as prodrugs. Tetraplatin (also named ormaplatin, (*OC*-6-22)-tetrachlorido(cyclohexane-1*R*,2*R*-diamine)platinum(IV)), iproplatin [(*OC*-6-33)-bis(hydroxido)dichloridodiisopropylamineplatinum(IV)], satraplatin [(*OC*-6-43)-bis(acetato)amminedichloridocyclohexylamineplatinum(IV)], and LA-12 [(*OC*-6-43)-bis(acetato)(1-adamantylamine)amminedichloridoplatinum(IV)] have progressed to preclinical studies and clinical trials, although none of them has been approved for clinical use [5–9]. Furthermore, the rational design of biaction or multiaction Pt(IV) prodrugs based on the insertion of biologically active ligands in the axial positions (generally through their carboxylate functionality), acting as auxiliary drugs (ideally synergistic with cisplatin), improves their targeting and cellular accumulation, generating or stimulating the immune response or antimetastatic activity, and often overcomes Pt chemoresistance [10–19].

In cisplatin-based Pt(IV) prodrugs, we prefer the terms *symmetric* when the two axial ligands are identical and *unsymmetric* when the two axial ligands are different, rather than *asymmetric* or *nonsymmetric* which implies that all symmetry elements have been lost. In the former case, the addition of identical axial ligands maintains the *C*_2v_ symmetry; in the latter situation, the two-fold axis and the symmetry plane containing the cisplatin unit are lost but the plane passing through the two axial ligands and bisecting the original square planar Pt(II) moiety remains. The point group is now lowered from *C*_2v_ to *C*_s_ and the molecule is still achiral. Unsymmetric Pt(IV) prodrugs (functionalized in one single axial position) have been reported to exhibit biological activity comparable to or higher than their symmetric (doubly functionalized) counterparts. The remaining axial position was occupied by OH^−^ or Cl^−^ ligands, which were often originally present in the Pt(IV) synthon. Undoubtedly, a successful prodrug of this kind was asplatin or platin-A [20, 21].

Probably, the most prominent example of this perception is so far represented by monochalcoplatin (Figure 1) that was extremely more active than its symmetric homologue chalcoplatin, despite its higher lipophilicity and the ability to deliver two active chalcone molecules from the latter (chalcone is an inhibitor of the p53 mouse double minute 2 pathway). For example, in the human ovarian cancer cell line A2780, monochalcoplatin showed an increase of up to 83 and 132 times in cytotoxicity compared to chalcoplatin and cisplatin, respectively. Interestingly enough, this outstanding activity was related to efficient (but undefined) transport-mediated cellular uptake, as well as an “instant” reductive activation process. Furthermore, in monochalcoplatin, the presence of the two equatorial chlorido ligands appears to be essential for its striking uptake since the oxaliplatin-based Pt(IV) analogue appears to be deprived of any transport mechanism [22, 23].

Indeed, during the study of the three simple cisplatin-based Pt(IV) complexes (*OC*-6-33)-diamminedichloridodihydroxidoplatinum(IV) (**1**), (*OC*-6-44)-acetatodiamminedichloridohydroxidoplatinum(IV) (**2**), and (*OC*-6-33)-diamminedichloridodiacetatoplatinum(IV) (**3**, Figure 1) [24, 25], all bearing axial ligands deprived of biological activity, unsymmetric complex **2** was unexpectedly more active than its symmetric counterparts **1**–**3**, and therefore, clarification of the overall scenario, in particular the possible difference in their cellular uptake mechanism, is required.

Molecules with reasonable lipophilicity are generally prone to cross the lipid bilayer membrane following the concentration gradient (energy-independent passive diffusion). Alternatively, membrane transporter and membrane channel proteins can support the uptake of drugs deprived of the physicochemical properties mentioned previously [26]. The coexistence of the two types of mechanisms for drug uptake is emphasized in several reviews [27, 28].

Membrane transporters are generally grouped in two families: ATP-binding cassette (ABC) and solute carrier (SLC). The SLC22 superfamily is divided into three subfamilies based on the nature of the substrate: organic cation (OCT), organic anion (OAT), and organic zwitterion (OCTN) transporters. In particular, OCT1 (SLC22A1), OCT2 (SLC22A2), and OCT3 (SLC22A3) were found to play a role in the transport of metal-based drugs [29].

For the prototypal anticancer metallodrug cisplatin, simple passive diffusion across the membrane was originally proposed as the only uptake mechanism, despite its low lipophilicity. However, several experimental results suggested that active or facilitated transport contributes to cisplatin uptake and is clearly involved in intrinsic or acquired resistance [30–32]. Several transporter proteins have been associated with cisplatin influx (with a variable level of evidence and positive or negative effects on overall chemotherapeutic efficiency): copper transporters, especially Ctr1 (SLC31A1), and, to a lesser extent, organic cation transporters, OCTs [29, 33–36]. Each OCT exhibits tissue-specific localization; for example, OCT1 is the main isoform in the liver, and OCT2 is predominantly expressed in the kidney, whereas OCT3 is widely distributed in many tissues. OCT2 is believed to be responsible for the nephrotoxicity induced by cisplatin. Furthermore, OCT1 and OCT2 are overexpressed in several colon cancer cells and appear to mediate selective cellular uptake and, hence, the cytotoxicity of oxaliplatin in colorectal carcinomas. Very recently, the role of volume-regulated anion channels VRACs, best known for their role in cell-volume homeostasis, has been postulated in the transport of cisplatin and its Pt(II) congeners [37].

Although dozens of papers are dealing with the mechanism of cellular accumulation of cisplatin and its homologues [32, 38, 39], very little is known about Pt(IV) prodrugs. According to experimental evidence and QSAR studies, cellular accumulation of most Pt(IV) compounds is strictly dependent on their lipophilicity, and this is a significant clue to passive diffusion [6, 40–45]. Furthermore, it was demonstrated by mass spectrometry that (*OC*-6-33)-diamminedichloridodiacetatoplatinum(IV) (**3**, Figure 1) is not the substrate of Ctr1 (unless reduced with ascorbic acid) through its possible interaction with the model transport proteins Mets7 and Mnk1 [36, 43, 46–50]. Furthermore, cisplatin uptake was deeply reduced in A2780 cells in which the Ctr1 copper transporter gene was silenced. In contrast, uptake of the very lipophilic Pt(IV) di-octanoato analogue of **3** in the same resistant cell line was almost unchanged and then apparently independent of Ctr1 expression [51]. Few reports are available in the literature that describe the uptake of clinically tested Pt(IV) prodrugs through OCTs: satraplatin was a modest OCT substrate in A2780 and HCT116 (human colon adenocarcinoma) cells [52], while ormaplatin exhibited a high affinity for these transporters in human embryonic kidney (HEK) cells, although less than oxaliplatin itself [53].

Importantly, Göschl et al. suggested the involvement of active or facilitated transport in the cellular uptake of a few Pt(IV) complexes, containing chlorides as leaving groups and methylated ethylene diamines as carrier groups in the equatorial plane, based on Pt accumulation experiments performed under hypothermic conditions [54]. Unfortunately, the difficulty in inducing profound ATP depletion in the adherent human colon adenocarcinoma SW480 cells employed did not allow to clearly differentiate between the two possible mechanisms: active and then energy-dependent or facilitated and then energy-independent transport.

In the present paper, the cytotoxic activity in a panel of seven human tumor cell lines, including a cisplatin-resistant subtype, of the three simple cisplatin-based Pt(IV) models **1**–**3** (Figure 1) is discussed with respect to its lipophilicity, membrane affinity, reduction potentials, and reduction kinetics. Additionally, their uptake has been evaluated under normal and hypothermic conditions and in the presence of selective inhibitors. Finally, the unusual uptake of **2** in the A2780 and A2780cisR cell lines was tentatively deciphered on the basis of the expression of the known cisplatin transporter genes (Ctr1, OCTs) studied by RT-PCR.

## 2. Materials and Methods

### 2.1. General Procedures

All chemicals (Sigma Aldrich-Merck or Alfa Aesar-Thermo Fisher Scientific, except for where they are otherwise specified) were used as received and without further purification. Complexes (*SP*-4-2)-diammine dichlorido platinum(II) (cisplatin) [55], (*OC*-6-33)-diamminedichloridodihydroxidoplatinum(IV) (**1**) [56], (*OC*-6-44)-acetatodiamminedichloridohydroxidoplatinum(IV) (**2**) [44], and (*OC*-6-33)-diamminedichloridodiacetatoplatinum(IV) (**3**) [57] were prepared and characterized according to previously published procedures. Complexes **1**–**3** were also obtained in their ^15^N-labeled form using the same synthetic procedure, except for the use of ^15^NH_3_-labeled cisplatin as starting material (see Supplementary Material) [8, 44, 58, 59].

### 2.2. Stability in Solution and Reduction with Cytosol

The stability in the solution of Complexes **1**–**3** (100 *μ*M) was studied by aging the compounds for 72 h at 37°C in the dark in the same cell culture medium employed for the A2780 cell line (i.e., RPMI 1640 medium added with 10% fetal bovine serum, FBS). The area of the chromatographic peaks of the Pt complexes was monitored in HPLC-MS, using a 70 : 30 mixture of 15 mM aqueous HCOOH and CH_3_OH as the mobile phase (see Section 2.3, settings *i*), for further instrumental details). ESI mass spectra, recorded using cone and capillary voltages of +30 V (positive ion mode) and 2.70 kV, were used to confirm the identity of the species.

To study the reduction of ^15^N-labeled Complexes **1**–**3**, fresh cytosol was obtained from about 2 × 10^7^ A2780 cells using the FractionPrep kit (BioVision, Milpitas, CA, USA) and following the manufacturer's instructions. The reduction behavior was monitored by means of [^1^H, ^15^N] HSQC (heteronuclear single quantum correlation) NMR measurements. In particular, the complexes were dissolved (3 mg) in a 9 : 1 mixture of cytosolic extract of A2780 cells and D_2_O (final volume of 500 *μ*L). [^1^H, ^15^N] HSQC spectra were recorded at 300 K on a Bruker Avance III NMR spectrometer operating at 500 MHz (^1^H) with the standard Bruker sequence hsqcetgpsiz (0.2 s acquisition time, 8 scans, 1.3 s relaxation delay, and 128 F_1_ points) [58, 60, 61].

### 2.3. Chromatographic Indexes

Retention times, *t*_R_, were measured on two columns: (i) a standard C_18_ column (Phenomenex Phenosphere-NEXT, 250 × 4.6 mm, 5 *μ*m) and (ii) an IAM.PC.DD.2 column (Regis, 10 cm × 4.6 cm, 10 *μ*m packing 300 Å pore size), prepared by covalently bonding cell-membrane-forming phospholipids to silica. On (i), a chromatogram was performed for each complex (0.25 mM) in a fixed eluent composition (30% methanol/70% aqueous 15 mM formic acid; flow rate 0.5 mL·min^−1^) using a Waters HPLC-MS instrument (equipped with an Alliance 2695 separation module, a 2487 dual lambda absorbance detector, and a 3100 mass detector). The UV-vis detector was set at 210 nm. In (ii), the mobile phase consisted of 20 mM ammonium acetate buffer at pH 6.9 and acetonitrile flowing at 1 mL·min^−1^. The samples were dissolved in the mobile phase in a concentration range of 50–100 *μ*g·mL^−1^. An HPLC Varian ProStar instrument equipped with a 410 autosampler, a PDA 335 LC detector, and Galaxie Chromatography Data System Version 1.9.302.952 was used.

The different *t*_R_s were used to calculate log *k*′, which is the logarithm of the retention factor *k*′ = (*t*_*R*_ − *t*_0_)/*t*_0_, where *t*_0_ is the column dead time (i.e., the *t*_*R*_ of KCl or citric acid used as the internal reference for (i) and (ii), respectively) [62–64].

In the case of IAM, the log *K*_*W*_ values were calculated by an extrapolation method. Log *k′* values were determined at a minimum of three different percentages of acetonitrile (*ϕ*) in the mobile phases (from 10 to 50%, v/v), and the intercept values of the linear relationships between log *k′* and *ϕ* values were assumed as log *K*_*W*_ [65, 66].

The polar surface area (PSA) of Complexes **1**–**3** has been calculated according to the previously published procedures [67].

### 2.4. Cell Cultures

The compounds under investigation were tested on a panel of commercial human cancer cell lines: ovarian endometrioid adenocarcinoma A2780 (ICLC HTL98008), its cisplatin-resistant variant A2780cisR (ECACC 93112517), colon carcinoma HCT 116 (ECACC 91091005), breast invasive ductal carcinoma MCF7 (ECACC 86012803), embryonal carcinoma NTERA-2 clone D1 (also known as NT2/D1, ICLC HTL97025), and lung adenocarcinoma A549 (ICLC HTL03001). Furthermore, a cell line derived from the pleural effusion of a patient with untreated malignant pleural mesothelioma (MPM) was added to the panel, namely, MM98 (sarcomatoid phenotype) [68]. Commercial cells were purchased from the European Collection of Authenticated Cell Cultures (ECACC, UK) or the Interlab Cell Line Collection (ICLC, Genoa, Italy), while the MM98 cell line was obtained from the Pathology Unit of the National Hospital of Alessandria (Italy).

The following media were used to grow the cells: RPMI 1640 (for A2780, A2780cisR, NT2/D1, and A549 cells), DMEM (for MCF7 cells), DMEM supplemented with nonessential amino acids (for MM98 cells), and McCoy's 5A (for HCT 116 cells). All media contained L-glutamine (2 mM) and were added with penicillin (100 IU·mL^−1^), streptomycin (100 mg·L^−1^), and 10% heat inactivated FBS. To maintain resistance in A2780cisR cells, cisplatin (1 *μ*M) was added to the medium every two passages.

Continuous treatments (CT) were carried out at 37°C in a humidified chamber with 5% CO_2_. Cisplatin was dissolved in a 0.9% w/v NaCl aqueous solution and brought to pH = 3 with HCl (final stock concentration 1 mM). The Pt(IV) Complexes **1**–**3** were dissolved in milliQ water (final stock concentration 5 mM) and stored at −20°C. The stock concentration was confirmed with inductively coupled plasma-mass spectrometry (ICP-MS) measurements. The mother solutions were diluted in a complete medium to the required concentration.

To assess the growth inhibition of the compounds under investigation, a cell viability test (i.e., the resazurin reduction assay) was used. In brief, 2–5 × 10^3^ cells per well (depending on the cell line) were seeded in black sterile tissue culture-treated 96-well plates. At the end of the treatment (72 h), viability was evaluated with 100 *μ*g·mL^−1^ resazurin (Acros Chemicals, France) in a fresh medium for 1 h at 37°C, and the amount of the reduced product, resorufin, was measured by fluorescence (excitation *λ* = 535 nm, emission *λ* = 595 nm) with a Tecan Infinite F200Pro plate reader (Tecan, Austria) [69]. In each experiment, cells were challenged with drug candidates at different concentrations, and the final data were calculated from at least three replicates of the same experiment performed in triplicate. The fluorescence of 8 wells containing a medium without cells was used as a blank. Fluorescence data were normalized to 100% cell viability for nontreated cells. The half-inhibiting concentration (IC_50_), defined as the concentration of the drug that reduces cell viability by 50%, was obtained from the dose-response sigmoid using Origin Pro (version 8, Microcal Software Inc., Northampton, MA, USA).

### 2.5. Cellular Accumulation

A2780 and A2780cisR cells were seeded in 10 mm Petri dishes and challenged with complexes under investigation (10 *μ*M) in a complete medium for 4 h. At the end of the exposure, cells were washed three times with PBS, detached from Petri dishes using 0.05% trypsin 1x + 2% EDTA (HyClone, Thermo Fisher), and harvested in a completely fresh medium. An automatic cell counting device (Countess®, Life Technologies) was used to measure the number and mean diameter of each cell necessary to calculate the mean cellular (spherical) volume.

For the analysis of Pt accumulation, cells were transferred to a borosilicate glass tube and centrifuged at 1100 rpm for 5 min at room temperature. The supernatant was carefully removed by aspiration, while approximately, 200 *μ*L of the supernatant was left to limit cell loss. Cellular pellets were stored at −80°C until mineralization.

The determination of the platinum content was performed by ICP-MS (Thermo Optek X Series 2). Mineralization was carried out by adding 70% w/w HNO_3_ to each sample (after defrosting), followed by incubation for 1 h at 60°C in an ultrasonic bath. Before the ICP-MS measurement, HNO_3_ was diluted to a final concentration of 1%. The instrument settings were optimized to produce the highest platinum sensitivity, and the most abundant isotopes of Pt and In (used as an internal standard) were quantified at *m/z* 195 and 115, respectively.

The quantity of Pt found in cells and normalized on cell number (cellular Pt accumulation) was expressed as ng of Pt per 10^6^ cells. The mean cellular volume, calculated from the actual mean cell diameter measured for each sample (about 12 *μ*m), was used to obtain the volume of 10^6^ cells and, finally, the intracellular Pt concentration. At treatment time zero, 100 *μ*L of the medium was taken from each sample to verify the extracellular Pt concentration. The ratio between intracellular and the actual extracellular concentrations is defined as the accumulation ratio, AR [70]. The Pt content in the control experiment, which characterizes any possible Pt contamination during the overall process, was statistically insignificant with respect to the cellular Pt accumulated during treatment, even in the case of A2780cisR.

Experiments with the inhibitors were carried out at 37°C as follows: (i) NaN_3_ + 2-deoxy-D-glucose (2DG): preincubation for 30 min in PBS with 25 mM NaN_3_ and 25 mM 2DG, followed by a 10 *μ*M CT with the Pt compounds in Earle's Balanced Salt Solution (EBSS) for 1 h; (ii) cimetidine (CMT): coincubation with 1.5 mM CMT and 10 *μ*M Pt compounds in EBSS for 1 h; (iii) 1-methyl-4-phenylpyridin-1-ium (MPP^+^): coincubation with 2.0 mM MPP^+^ and 10 *μ*M Pt compounds in EBSS for 1 h; (iv) cytochalasin D (CTD): preincubation for 30 min in EBSS with 10 *μ*M CTD, followed by a 10 *μ*M CT with the Pt compounds in EBSS for 1 h; (v) O-benzylserine (BS): coincubation with 1.0 mM BS and 10 *μ*M Pt compounds in EBSS for 4 h; (vi) ouabain (OUA): preincubation for 1 h in EBSS with 1 mM OUA, followed by a 10 *μ*M CT with the Pt compounds in EBSS for 4 h; (vii) tetraethylammonium chloride (TEA): preincubation for 1 h with 2.0 mM TEA followed by 10 *μ*M Pt compounds in EBSS for 4 h. In the experiments at low temperature, A2780 cells were preincubated for 2 h at 4°C, followed by CT with each Pt compound (10 *μ*M) for 4 h. In all cases, at the end of the CT, the cells were manipulated as described in the previous paragraphs.

### 2.6. DNA Platination

Cells were seeded in 175 cm^2^ flasks and treated for 24 h with the complexes under investigation (10 *μ*M) as described previously. From this sample, about 5 × 10^6^ cells were taken out for the cellular accumulation analysis (see the previous section), whereas about 20 × 10^6^ cells were taken out for the DNA platination analysis.

For DNA platination analysis, cells were transferred to a plastic tube and centrifuged at 1100 rpm for 5 min at room temperature. The supernatant was carefully removed by aspiration, and the cell pellets were stored at −20°C until total genomic DNA was extracted with a commercial kit (PerfectPure Cultured Mammalian Cells, 5 Prime-Eppendorf), following the manufacturer's instructions. In brief, during cell lysis, DNA was purified by treatment with RNAse A and proteinase K and then extracted on silica-based centrifugation columns.

After washing, DNA was eluted in 300 *μ*L of elution buffer. An amount of 8 *μ*L of sample or elution buffer (used as blank) was diluted in TE buffer (10 mM Tris-HCl, 1 mM EDTA, and pH 8.0) to 80 *μ*L, corresponding to a path length of 0.5 cm in UV-transparent microplate half-area wells (UVStar®, Greiner Bio-One). Absorbance at 260 nm (*A*_260_, relative to nucleic acids) and 280 nm (*A*_280_, relative to proteins) was recorded from triplicate wells.

For each well, *A*_260_ and *A*_280_ were corrected by subtracting the background, and then the purity of the samples was verified by means of the *A*_260_ to *A*_280_ ratio. After subtracting the mean *A*_260_ from the blank wells, the DNA concentration was calculated from the corrected *A*_260_ using a calibration curve obtained with the calf thymus DNA. Under these conditions, an absorbance of 1 unit at 260 nm corresponds to 100 *μ*g of DNA per mL. The remaining amount of DNA elution buffer was transferred to a borosilicate glass tube, and its precise volume was determined by weight to calculate the total amount of DNA and then stored at −20°C until mineralization.

The amount of Pt bound to DNA (experimental *P*_DNA_) was expressed as pg of Pt per *μ*g of DNA experimentally found. Taking into account that one million human female diploid nuclei contain 6.55 *μ*g of DNA, the total amount of DNA-bound Pt per million cells was calculated by multiplying the experimental *P*_DNA_ by 6.55 [43]. Therefore, the percentage of intracellular Pt bound to DNA (*P*_DNA_%) is defined as the ratio between platination and cellular accumulation, corrected by the factor related to the cellular content of DNA:

(1) $$\begin{array}{c} P_{DNA}\%=\frac{P_{DNA}\times 6.55}{1000\times cellular\;accumulation}\times 100\text{.} \end{array}$$

### 2.7. Caspase 3 Activity

A2780 cells (20 × 10^3^) were seeded in 96-well TC plates the day before treatment, which was performed with 10 *μ*M concentrations of Pt compounds. After 24 h, some pictures were taken using an inverted contrast phase microscope (Leica DMIL LED). Cells were washed with EBSS and lysed on ice with 25 *μ*M lysis buffer (10 mM HEPES, 2 mM EDTA, 2 mM DTT, 0.1% CHAPS, and pH 7.4). The caspase 3/7 inhibitor Ac-DEVD-CHO (*N*-Ac-Asp-Glu-Val-Asp-CHO) was added to the control wells at a concentration of 0.01 g·L^−1^. Then, 200 *μ*L of the caspase-3 fluorescent substrate, Ac-DEVD-AFC (*N*-Acetyl-Asp-Glu-Val-Asp-7-amino-4-trifluoromethylcoumarin, 0.01 g·L^−1^ in lysis buffer), was added to all wells, and mixed and 200 *μ*L of each sample was transferred to a black microtiter plate. The activity was followed for 1 h, by means of fluorescence at excitation *λ* = 390 nm, emission *λ* = 520 nm, normalized over the blank [71]. The final fold activity was calculated with respect to the control wells and normalized with respect to the residual viability.

### 2.8. RNA Isolation and RT-PCR

Total RNA was extracted using the guanidinium thiocyanate method [72]. Starting from equal amounts of RNA, cDNA was synthesized by reverse transcription reaction using the RevertAid Minus First Strand cDNA Synthesis Kit (Fermentas-Thermo Scientific, Burlington, Canada), using random hexamers as primers, according to the manufacturer's instructions. An amount of 20 ng of cDNA was used to perform RT-PCR amplification of mRNA. The primers for Ctr1 were 5′-CTGCTGCGTAAGTCACAAGTCAG-3′ (forward), 5′-TATGACCACCTGGATGATGTGC-3′ (reverse); for OCT1: 5′-ACTCCGCTCTGGTCGAAATC-3′ (forward), 5′-CGACATCGCCGCAAAACAT-3′ (reverse); for OCT2: 5′-ACTCTGCCCTGGTTGAATTC-3′(forward), 5′-GCAACGGTCTCTCTTCTTAG-3′ (reverse); for OCT3: 5′-CAGAGATCACTGTTACAGAT-3′ (forward), 5′-GATAGCTCCTTCTTTCTGTC-3′ (reverse). 18S rRNA was used as the reference gene.

## 3. Results and Discussion

### 3.1. Antiproliferative Activity and Physicochemical Parameters

Based on its structure, which is intermediate between those of **1** and **3** (Table 1), **2** is expected to be rather ineffective as an antiproliferative agent, as its congeners are. However, **2**, included in routine viability tests carried out in our laboratory, surprisingly exhibited a remarkable potency (10–100 times higher than those of **1** and **3**) against a panel of human cancer cell lines (Table 2). The average IC_50_ of **2** (2.91 *μ*M) was roughly similar to that of cisplatin (2.72 *μ*M) in the wild cell panel. Furthermore, **2** was partially able to bypass chemoresistance in the cisplatin-resistant A2780cisR cell line, being the resistance factor RF (**2**) = 6 *vs.* RF (cisplatin) = 13 (RF = IC_50_ A2780cisR/IC_50_ A2780).

The IC_50_ data of **2** on all the cell lines employed represent an anomaly if we reason in terms of passive diffusion only, which is modulated by lipophilicity. In fact, the lipophilicity of **2** is expected to be intermediate between those of **1** and **3**, simply by comparing the chemical structures of the three complexes.

The lipophilicity of a compound is commonly described by the octanol-water partition coefficient, log *P*_*o*/*w*_, which can be defined as the ratio of the concentration of the compound between the organic (*n*-octanol) and the aqueous phase. However, other faster methods, such as RP-HPLC, have often been used to evaluate lipophilicity instead of the shake-flask method. In this case, retention is due to partitioning between the lipophilic C_18_ chains of the stationary phase and the aqueous eluent [73, 74]. Therefore, the retention times (*t*_*R*_) of the complexes were determined on a C_18_ column using a 70/30 (% v/v) water/MeOH mixture as an eluent, and the data were expressed as log *k*′ (*k*′ = (*t*_*R*_ − *t*_0_)/*t*_0_, where *t*_0_ is the column dead time of the column). The trend of the measured log *k'* was **3** > **2** > cisplatin > **1**, as expected based on the structure of the complexes, the acetato ligand being more lipophilic than the hydroxido one (Table 1).

Immobilized artificial membrane (IAM) chromatography is a complementary technique for evaluating the interaction of a drug with the cellular membrane. In this technique, the stationary silica phase is covalently bonded to phospholipids, giving a material that is more similar to the cell membrane than the simpler aliphatic chains of the RP-HPLC columns. The log *K*_*W*_ values (i.e., log *k′* values extrapolated at fully aqueous mobile phase) show the same trend for the three Pt(IV) complexes (log *K*_*W*_ = −0.64, −1.06, −0.96, and −0.83 for cisplatin, **1**, **2**, and **3**, respectively) (Table 1).

The polar surface area (PSA), defined as the surface sum over all polar atoms, including attached hydrogen, can be used as a descriptor of the polar component of lipophilicity. Generally speaking, compounds with PSA ≥140 Å^2^ should exhibit poor cell penetration (≤10%), whereas compounds with PSA ≤60 Å^2^ show high absorption (≥90%) [75]. The calculated PSA values obtained for the **1**–**3** compounds (116, 112, and 110 Å^2^, respectively) confirm, albeit on a different scale, the previously observed trend (Table 2). Thus, intermediate cellular accumulation, then intermediate DNA platination, and then intermediate antiproliferative potency are predictable from these comparisons.

Being prodrugs, Pt(IV) complexes must be activated *in vivo*, their reduction potential can offer information on the feasibility of the reduction process, especially in the case of an outer-sphere mechanism [5, 6, 76]. However, this may not be sufficient to guarantee *in vivo* reduction, as the kinetic characteristics of chemical reduction play an important role, especially in the case of an inner-sphere mechanism [8, 9]. Interestingly enough, the peak potential (*E*_*p*_) trend is opposite to that of kinetics (*k*, see the *t*_½_ values in Table 1). Indeed, these Pt(IV) derivatives are prone to electrochemical reduction in the order **1** < **2** < **3** but susceptible to chemical reduction with monoascorbate (the form of ascorbic acid mainly present at physiological pH) in the order **1** > **2** > **3**. The presence of OH ligands, while rendering the electrochemical potential more negative (less favorable to outer-sphere reduction), represents an efficient bridge for inner-sphere electron transfer. The overall scenario is in agreement with what Wexselblatt and Gibson observed for similar complexes [8, 77]. Although for **1**–**3**, the trend of the two parameters (*E*_*p*_ and *k*) is opposite, complex **2** exhibits an intermediate value on both scales.

Therefore, to shed light on the possible influence of the reduction process on the biological activity of **1**–**3**, their stability towards reduction was studied directly in the cytosol extract of the cells under investigation [44, 56, 58, 60, 64, 78, 79]. In fact, ^15^N-labeled complexes, ^15^**N-1**, ^15^**N-2**, and ^15^**N-3**, were directly challenged in the dark with cytosol extracted from A2780 cells, and their reduction was monitored by ^15^N NMR spectroscopy (see Figures S2–S9, Supplementary Material). Actually, ^15^N NMR spectroscopy provides information on the oxidation state of the metal and the nature of the donor atoms in the coordinated ligands, allowing faster and more sensitive analyses than ^195^Pt NMR. The signals of the synthesized ^15^N-labeled ^15^**N-1**, ^15^**N-2**, and ^15^**N-3** complexes exhibit chemical shifts between −30 and −40 ppm [78, 80]. After approximately 2 h, in the [^1^H, ^15^N] HSQC spectra, the original signals of the Pt(IV) complex almost disappeared (^15^**N-1**^15^NH_3_* δ* = –33.8 ppm and ^1^H *δ* = 5.6 ppm; ^15^**N-2**^15^NH_3_* δ* = −35.6 ppm and ^1^H *δ* = 6.0 ppm; ^15^**N-3**^15^NH_3_* δ* = –39.6 ppm and ^1^H *δ* = 6.4 ppm). Conversely, the new ^15^NH_3_ peak at *δ* = –66.7 ppm and the ^1^H peak at *δ* = 4.0 ppm (^1^*J*_Pt-N_ = 331 Hz and ^2^*J*_Pt-H_ = 63 Hz) can be assigned to cisplatin. Moreover, other weaker signals were present in the chemical shift range of Pt(II) complexes, corresponding to one ^15^N *trans* to Cl and one ^15^N *trans* to oxygen. Such signals indicate the formation of aquated Pt(II) species (see Supplementary Material) [78].

Since the effective reduction time of **1**–**3** (2 h) is negligible with respect to the schedule for the evaluation of IC_50_ (72 h), the different cytotoxicity of **2** cannot be explained in terms of the different reduction speeds with respect to **1** and **3**, differences often invoked for other cisplatin-based Pt(IV) conjugates [23].

At this stage, all the experimental data seem to point to an additional mechanism of uptake other than passive diffusion for complex **2** only. For this reason, several experiments were designed to highlight the possible role of cell uptake in the anomalous cytotoxicity observed.

### 3.2. Uptake Experiments on A2780 Cells

Among the panel of tumor cells used in viability tests, the human ovarian endometrioid adenocarcinoma cell line A2780 was chosen for further mechanistic investigation, as it has frequently been used to test the cytotoxicity of several platinum complexes. Furthermore, the A2780 line was very sensitive to **2**, and its cisplatin-resistant subline (A2780cisR) is commercially available. Finally, OCTs (albeit to a different degree) are expressed (to some extent) in such a cell line as in many ovarian tumors [52]. Interestingly, Ctr1 expression was reported to be significantly reduced in A2780cisR cells compared to their wild-type sensitive counterparts [81].

**Figure 2:**
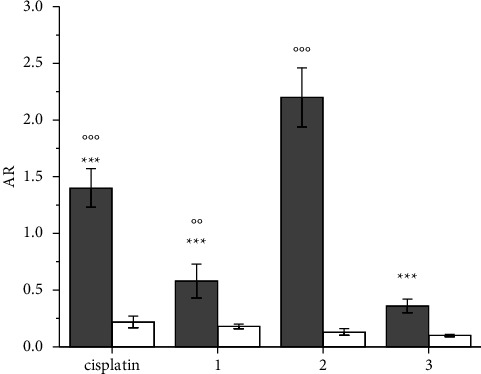
Accumulation ratio (AR) of cisplatin and **1**–**3** complexes in A2780 cells treated for 4 h at 37°C (gray bars) and 4°C (white bars) with 10 *μ*M concentrations of the compounds. Data are the mean ± sd of three independent replicates and were compared using one-way analysis of variance (ANOVA) with the Tukey post-hoc test. Statistical analysis (cisplatin, 1, and 3 *vs.* 2, 37°C): ^*∗∗∗*^*p* < 0.001; (37°C *vs.* 4°C): °°*p* < 0.01; °°°*p* < 0.001.

Cellular accumulation of cisplatin and **1**–**3** was evaluated using the ICP-MS technique after 4 h of CT at 37°C and expressed as the accumulation ratio (AR, that is, the ratio between the intracellular and extracellular Pt concentrations) (Figure 2, gray bars).

Complex **2** exhibited an unexpected (based solely on lipophilicity) higher AR at 4 h than **1** and **3**, even higher than that of cisplatin, indicating that passive diffusion is not the discriminant for remarkably different uptake. Interestingly enough, cisplatin also showed an AR higher than predicted simply on the basis of passive diffusion due to its low lipophilicity, but this was probably due to the contribution of the additional Ctr1 transport mechanism [36, 50, 82].

**Figure 3:**
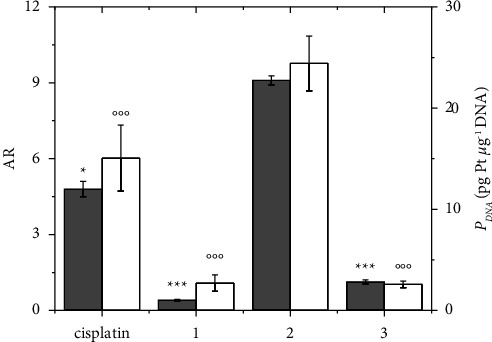
AR (gray bars, left axis scale) and DNA platination (*P*_DNA_ in pg Pt *μ*g^−1^ DNA, white bars, right axis scale) of cisplatin and Complexes **1**–**3** in A2780 cells after 24 h CT at 10 *μ*M concentration. Data are the mean ± sd of three independent replicates and were compared using one-way analysis of variance (ANOVA) with the Tukey post-hoc test. Statistical analysis (AR, cisplatin, **1**, and **3***vs. ***2**): ^*∗*^*p* < 0.05, ^*∗∗∗*^*p* < 0.001; (*P*_DNA_, cisplatin, **1**, and **3***vs. ***2**): °°°*p* < 0.001.

The same experiment was repeated for prolonged treatment (24 h) to reach detectable levels of DNA platination necessary for comparison purposes, obtaining a similar trend (Figure 3) [57, 83]. DNA platination carried out by **2** was again the highest among Complexes **1**–**3** and was similar to that of cisplatin, paralleling the highest uptake and explaining the antiproliferative activity; thus suggesting that once internalized and reduced in cytosol, the reactivity of all cisplatin-based Pt(IV) complexes is identical to that of metabolite cisplatin [84, 85].

### 3.3. Induction of Apoptosis

**Figure 4:**
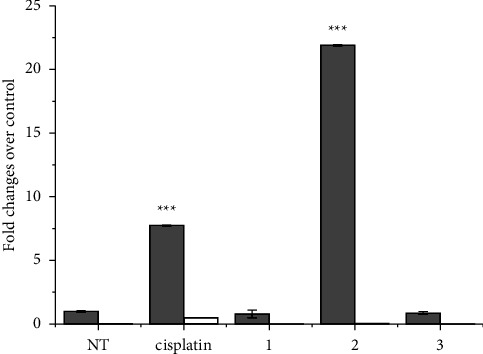
The caspase 3/7 activity assay. A2780 cells were treated with cisplatin and **1**–**3** at equimolar concentrations (10 *μ*M) for 24 h CT. The Ac-DEVD-AFC fluorogenic substrate of caspase 3/7 was added to treated cells to highlight caspase activity (gray bars). Parallelly, the Ac-DEVD-CHO inhibitor was also added to a second set of treated cells to verify the specific activity (white bars, visible only for cisplatin). Data are the mean ± SD of three independent replicates and were compared using one-way analysis of variance (ANOVA) with the Tukey post-hoc test. Statistical analysis (cisplatin, **1**–**3***vs.* NT): no indication = not significant, ^*∗∗∗*^*p* < 0.001.

The cytotoxic effect of cisplatin is mainly related to the formation of intrastrand and interstrand DNA cross-links carried out by the active electrophilic agent [Pt(NH_3_)_2_]^2+^, which distorts the structure of DNA and inhibits replication and transcription, finally triggering apoptosis. The cysteinyl aspartate specific protease (caspase) cascade is activated during apoptosis induction [86, 87]. Therefore, the activity of caspases 3/7 was investigated after 24 h CT of A2780 cells with 10 *μ*M concentrations of cisplatin or **1**–**3**. The increase in caspase 3/7 activity followed the same trend of Pt accumulation and DNA platination (Figure 4), indicating that **1**–**3** operate with a similar DNA-damaging mechanism as its metabolite cisplatin, finally bringing cells to apoptosis.

### 3.4. Attempts to Clarify the Uptake Mechanism

As already reported in the Introduction, molecules with reasonable lipophilicity are generally prone to cross the lipid bilayer membrane by simple energy-independent passive diffusion. Alternatively, membrane transporters and membrane-channel proteins can support the internalization of drugs deprived of the physicochemical properties mentioned previously, allowing efficient cell uptake [26]. The coexistence of the two mechanisms for drug uptake is emphasized in several reviews [27, 28]. The reason behind the anomalous cellular accumulation of **2** remains a puzzling question. However, the results obtained so far point to the involvement of an active/facilitated transport that is complementary/alternative to passive diffusion. To shed light on this idea, analysis of the cellular accumulation at low temperature or in the presence of a set of inhibitors of any likely uptake mechanisms was attempted.

Carrier-mediated transport, whether active or facilitated, is strongly affected by decreasing temperatures. Therefore, the cellular accumulation of **2** along with cisplatin **1** and **3** was analyzed after 4 h CT under hypothermic conditions (4°C) (Figure 2) and compared with that at 37°C. As expected, AR decreased for all compounds but to a greater extent for cisplatin and, especially, for **2**. In fact, the ratio between AR 37°C and AR 4°C is 3.2 and 3.6 for **1** and **3** but increases hugely for cisplatin and especially for **2**, indicating that active/facilitated transport contributes significantly to the overall uptake of the latter two complexes.

**Figure 5:**
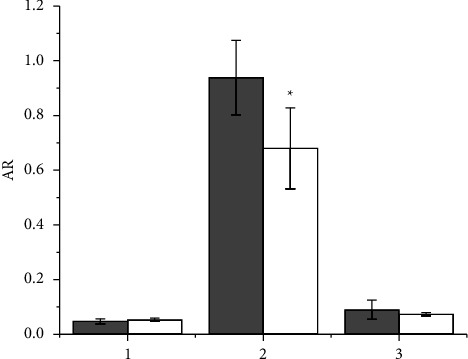
AR in A2780 cells treated for **1** h with **1**, **2**, and **3** (10 *μ*M) in the absence (gray bar) or in the presence (white bar) of NaN_3_ and 2-deoxyglucose (NaN_3_/2DG, pretreatment, both 25 mM). Data are the mean ± SD of three independent replicates and were compared using one-way analysis of variance (ANOVA) with the Tukey post-hoc test. Statistical analysis (treated *vs.* untreated): no indication = not significant, ^*∗*^*p* < 0.05.

Energy-dependent transport is inhibited by the decrease in the ATP level that can be achieved by pretreatment with 2-deoxyglucose (2DG) and NaN_3_. To limit the toxicity of such a pretreatment that could compromise the viability of the cells under investigation, a set of preliminary experiments was carried out to find the optimal concentration of inhibitors and the timing of the pretreatment capable of preserving cell adhesion, even at lower levels of ATP levels (data not shown). The NaN_3_/2DG pretreatment employed (see Materials and Methods) statistically reduced the cell accumulation of **2** (Figure 5), without appreciably affecting those of **1** and **3**.

**Figure 6:**
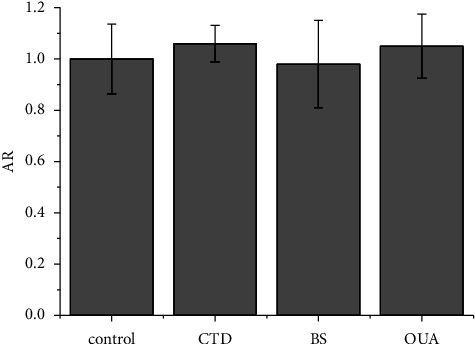
AR in A2780 cells treated for 1 h with **2** (10 *μ*M) in the absence (control) or in the presence of inhibitors cytochalasin D (CTD pretreatment, 10 *μ*M), O-benzylserine (BS, cotreatment, 1 mM), or ouabain (OUA, pretreatment, 1 mM). Data are the mean ± SD of three independent replicates and were compared using one-way analysis of variance (ANOVA) with the Tukey post-hoc test. Statistical analysis (inhibitors *vs.* controls): no indication = not significant.

Since the cellular accumulation of **2** was energy-dependent, endocytosis (i.e., engulfment of the plasma membrane to trap extracellular substances in the vesicles) was first considered, although **2** is a low *M*_*r*_ molecule. Since endocytosis is due to the ATP-dependent polymerization of actin microfilaments, it can be inhibited by cytochalasin D (CTD) [88, 89]. This inhibitor did not cause any decrease in Pt accumulation of **2** (Figure 6). The same was true for **1** and **3** (data not shown). A similar negative response was previously found for the larger monochalcoplatin molecule (Figure 1) using wortmannin as an inhibitor of endocytosis and micropinocytosis [23]. These data suggest that the abnormal high cell accumulation of **2** is based on carrier-mediated transport.

Depending on its direction (along or against the electrochemical gradient), carrier-mediated transport can be passive or active, with the latter transport dependent on the ATP or ion gradient. A well-known carrier of zwitterions is the glutamine transporter ASCT2 (SLC1A5), which exchanges extracellular glutamine for Na^+^, and can be inhibited by O-benzylserine (BS) [90]. Alternatively, ouabain (OUA) lowers the Na^+^ gradient by inhibiting Na^+^/K^+^ ATPase. The two inhibitors did not cause a significant effect on the cellular accumulation of **2** (Figure 6), thus excluding the direct involvement of Na^+^ and K^+^ gradients.

**Figure 7:**
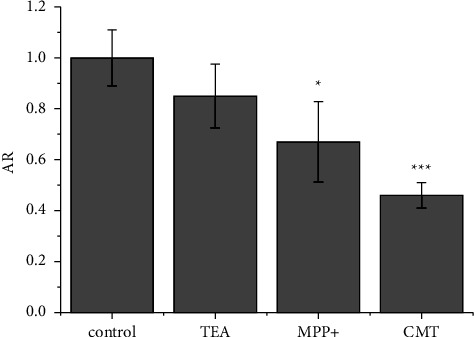
AR in A2780 cells treated for 1 h with **2** (10 *μ*M) in the absence (control) or in the presence of tetraethylammonium chloride (TEA, pretreatment, 20 mg·mL^−1^), or 1-methyl-4-phenylpyridin-1-ium (MPP^+^, cotreatment, 2 mM), or cimetidine (CMT, cotreatment, 1.5 mM). data are the mean ± SD of three independent replicates and were compared using one-way analysis of variance (ANOVA) with the Tukey post-hoc test. Statistical analysis (inhibitors *vs.* controls): no indication = not significant, ^*∗*^*p* < 0.05, ^*∗∗∗*^*p* < 0.001.

Among several remaining candidates for such transport, OCTs appear to be possible ones on the basis of data from the literature [29–32, 34, 36, 91]. OCTs mediate the electrogenic and sodium-independent translocation of organic cations across the membrane in either direction, being the driving force of the electrochemical gradient and the inside-negative membrane potential [92]. Thus, their activity is not directly dependent on ATP but can be markedly reduced when metabolic inhibition occurs [93]. OCTs recognize hydrophilic substrates such as tetraethylammonium (TEA), the prototype small organic cation, and, to a greater extent, bulkier molecules, such as the neurotoxin 1-methyl-4-phenylpyridin-1-ium (MPP^+^) and the H_2_-receptor antagonist cimetidine (CMT) [94, 95]. Actually, TEA produced an evident (albeit statistically not significant) decrease in the uptake of **2**, whereas MPP^+^ and to a greater extent CMT inhibited the cell accumulation of **2** (Figure 7). These data indicate that OCTs are possible carrier proteins for the uptake of **2**. In contrast, AR values of **1** and **3** were not significantly affected by the presence of such OCT inhibitors (see Figure S10, Supplementary Material, for the comparison of uptake of **1**–**3** in the presence of the most efficient inhibitor, CMT).

So far, few structure-activity relationships on OCT substrates are available; these substrates generally contain a hydrophobic interaction domain in combination with an ionizable site [96, 97]. In the case of the few examples regarding Pt(IV) prodrugs, one can observe that OCTs mediate the uptake of complexes containing an organic portion as an equatorial nonleaving group (i.e., the cyclohexane-1,2-diamine in ormaplatin and the cyclohexylamine in satraplatin) in combination with a hydrolyzable site (the chlorido equatorial leaving ligands of both complexes) able to generate a transient positive charge by aquation. Because of the high positive charge of Pt(IV), the final products of hydrolysis are inevitably the mono- and eventually the dihydroxido species. Indeed, Kastner et al. aged seven diacetato Pt(IV) complexes at 37°C for 24 h in phosphate buffer at different pH values. On this timescale of the experiment, most of the satraplatin and oxaliplatin-based complexes studied were hydrolyzed, with the formation of mono and di-hydroxido species. On the contrary, the cisplatin and carboplatin derivatives remained almost unchanged [98]. Interestingly enough, the increasing *σ*-donor ability of the equatorial nonleaving N-ligands (NH_3_ < NH_2_R < NHR_2_) *trans* to chlorides improves such a hydrolysis [98].

The solution behavior of **1**–**3** was investigated by aging the compounds in the dark for 72 h in the same cell culture medium used for the A2780 cell line (i.e., RPMI 1640 medium added with 10% fetal bovine serum), monitoring the HPLC peak area. Under these conditions, **1** (>95% of the original HPLC peak) and **3** (>85% of the original HPLC peak) remained almost intact (see Figure S11, Supplementary Material), whereas **2** underwent partial hydrolysis (approximately 40%, see Figure S12, Supplementary Material).

The MS spectrum associated with the new HPLC peak of **2** corresponds to the protonated [PtCl(NH_3_)_2_(OH)(OH_2_)(O_2_CMe)]^+^ ion, indicating the successful aquation of an equatorial chloride in the unsymmetric complex (see Figure S12, Supplementary Material). Similar behavior, even if to a minor extent, was observed for complex **3**. This hydrolysis generates (albeit partially and transiently) a cationic complex that may be a suitable substrate for OCTs. Given the variety of components of the complete RPMI 1640 medium (amino acids, proteins, inorganic salts, vitamins, etc.), it is difficult to suggest which is the promoting agent of such a hydrolysis in **2** and **3** [99]. In addition, FBS contains esterases [100–102], in particular carboxylesterases, which are potentially able to promote the hydrolysis of carboxyl groups, i.e., the axial acetato ligand/s bound to the Pt(IV) center in **2** and **3**. This would involve the formal substitution of a CH_3_COO^−^ group with an HO^−^ group, thus turning **3** into **2** and **2** into **1**. HPLC profiles (where the retention times of Complexes **1**–**3** and their hydrolyzed species are distinct) combined with ESI-MS spectra do not show evidence of such behavior under the experimental conditions used. Possible trace species may be covered by the background of peaks corresponding to the medium.

**Figure 8:**
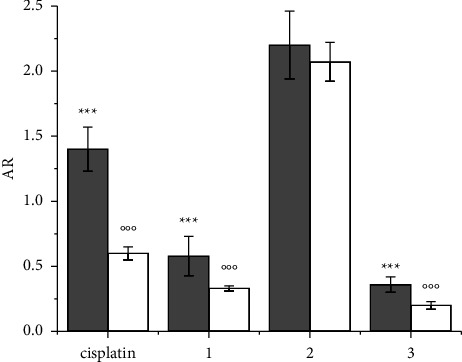
AR of cisplatin and **1**–**3** in A2780 (wild type; gray bars) and A2780cisR (cisplatin-resistant; white bars) cells after 4 h CT at a 10 *μ*M concentration of compounds. Data are the mean ± SD of three independent replicates and were compared using one-way analysis of variance (ANOVA) with the Tukey post-hoc test. Statistical analysis (cisplatin, **1** and **3***vs. ***2**, A2780): ^*∗∗∗*^*p* < 0.001; (cisplatin, **1** and **3***vs. ***2**, A2780cisR): °°°*p* < 0.001.

**Figure 9:**
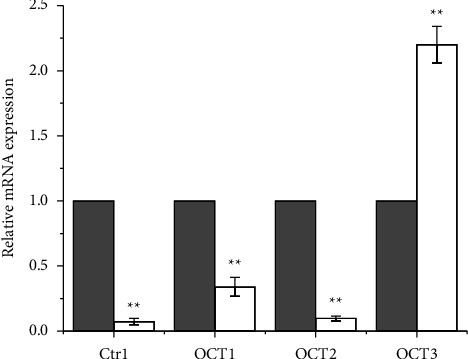
Expression of Ctr1, OCT1, OCT2, and OCT3 mRNA in A2780cisR (cisplatin resistant; white bars) cells compared to A2780 (wild type; gray bars). Data are the mean ± SD of three independent replicates and were compared using one-way analysis of variance (ANOVA) with the Tukey post-hoc test. Statistical analysis (A2780cisR *vs.* A2780): ^*∗∗*^*p* < 0.01).

In an attempt to clarify whether this transport mechanism plays a role in the partial circumvention of resistance shown by **2** (Table 2), the AR values of **1**–**3** and cisplatin as a reference were evaluated on both wild-type A2780 and its cisplatin-resistant subline A2780cisR (Figure 8). After 4 h CT with cisplatin, A2780cisR cells accumulated much lower amounts of Pt than A2780, as expected. This resistance was traced back to the lower expression of Ctr1, as reported in the literature [67], and the experiment summarized in Figure 9 confirms this view. The same behavior is shown in **1** and **3** (Figure 9). On the contrary, in both cell lines, **2** showed similar AR levels. A more efficient passive diffusion (the usual reason why lipophilic Pt(IV) prodrugs, deprived of biologically active axial ligands, bypass chemoresistance due to decreased cellular uptake [103]) cannot be invoked for **2** since its values of log *k*′ and log *K*_*W*_ are not very different from those of cisplatin, **1** and **3**.

Thus, the reason for the almost unaltered uptake of **2** in A2780cisR cells with respect to the wild-type counterparts could be that **2** benefits of some carriers for its energy-dependent cellular uptake. For this reason, the expression of OCT1, OCT2, and OCT3 mRNA was evaluated in cisplatin-resistant A2780cisR and wild-type A2780 cells, as well as Ctr1, albeit Ctr1 was previously discarded as a Pt(IV) prodrug carrier [43]. As shown in Figure 9, Ctr1, OCT1, and OCT2 were lower in A2780cisR than in A2780, whereas OCT3 was significantly higher. This scenario is not very different from that found for the expression of A2780 cells made resistant to the Au-based metal-drug auranofin [104]. This behavior suggests a role for OCT3 in the transport of **2** in A2780cisR. The overcome of chemoresistance is only partial, being 6 the RF value of **2** compared to 13 for cisplatin. Other mechanisms in the multifactorial phenomenon of chemoresistance could play a role in addition to different uptake efficiencies [105]. Only highly lipophilic Pt(IV) prodrugs, which have an enormously higher cellular uptake than cisplatin, are able to almost completely overcome resistance [51].

## 4. Conclusions

The unexpectedly higher cytotoxicity of unsymmetric **2** (comparable to that of cisplatin) with respect to its symmetric homologues **1** and **3**, having similar structure, reduction propensity, and lipophilicity, in the human tumor cell lines investigated, is due to its peculiar cellular accumulation. Indeed, the increased intracellular accumulation of **2** parallels the increased DNA platination and the increased induction of apoptosis.

**Figure 10:**
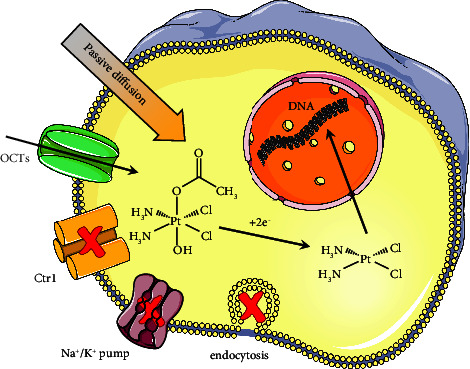
A tentative scheme of the suggested mechanism of internalization of compound **2**.

Complex **2** revealed an energy-dependent uptake mechanism, additional/alternative to the passive diffusion typical of all Pt(IV) prodrugs. Although the exact mechanism of cellular uptake cannot be deciphered by inhibition experiments alone, OCTs are possible transporter proteins (Figure 10). In particular, OCT3 seems to be the most probable candidate among the transporters considered being the only one upregulated in A2780cisR. Interestingly, MCF7 cells, devoid of any OCT3 expression [106], show the same low sensitivity to **2** as cisplatin does (Table 2).

A better understanding of such uptake could be achieved only by employing specific cell lines engineered for over-expression or under-expression of any possible gene of carrier proteins.

Indeed, **2** does not represent the lead candidate for Pt(IV) anticancer prodrugs, as dozens of lipophilic and multifunctional analogues exhibit much higher cytotoxic activity (in the nM range) [16]. However, its particular behavior offers an unexpected example of active transport within the family of Pt(IV) complexes, a possible mechanism that should be taken into account in any future design of such promising agents.

## Figures and Tables

**Table 1:** Lipophilicity (HPLC log *k*′ measured on a C_18_ column and log *K*_*W*_ measured on the immobilized artificial membrane column), polar surface area (PSA), half-life (*t*_½_ at 37°C in the presence of a ten-fold excess of ascorbic acid), and reduction peak potential (*E*_*p*_, V *vs.* Ag/AgCl, 3M KCl, glassy carbon electrode, and the scan rate 0.2 V·s^−1^) for Complexes **1**–**3**.

Compounds	Log *k′*	Log *K*_*W*_	PSA (Å^2^)	*E*_*p*_ (V)^a^	*t*_½_ (min)^a^
**1**	−1.50	−1.06	116	−0.815	8.6
**2**	−1.10	−0.96	112	−0.618	20.5
**3**	−0.84	−0.83	110	−0.486	528

^a^ from [25].

**Table 2:** Antiproliferative data (IC_50_, *μ*M) for Complexes **1**–**3** obtained after 72 h of treatment. Data in brackets represent the resistance factor, RF = IC_50_ A2780cisR (cisplatin-resistant)/IC_50_ A2780 (wild-type), rounded to the nearest unity. Data are the mean ± standard deviation (sd) of at least three replicates.

Compound	IC_50_ (*μ*M)						
	A2780	A2780cisR	NT2-D1	HCT 116	A549	MCF-7	MM98
Cisplatin	0.48 ± 0.12	6.22 ± 1.02 (13)	0.10 ± 0.04	2.30 ± 0.30	3.80 ± 0.70	6.46 ± 0.91	3.20 ± 1.02
**1**	3.71 ± 1.93	52.11 ± 3.48 (14)	1.44 ± 0.16	22.95 ± 3.89	48.20 ± 27.70	78.10 ± 5.60	>100
**2**	0.16 ± 0.05	1.02 ± 0.68 (6)	0.21 ± 0.08	1.46 ± 0.02	3.11 ± 0.74	8.86 ± 3.45	3.67 ± 1.32
**3**	12.10 ± 5.20	>100	4.30 ± 1.13	55.26 ± 1.69	77.98 ± 17.66	>100	>100

## Data Availability

All data supporting the results are included within the article and in the Supplementary Materials.

## Supplementary Materials

Supplementary Materials Figure S1: sketch of the complexes under investigation. Figures S2–S3: [^1^H, ^15^N] HSQC spectrum of ^15^**N-1** before and after 2 h reduction in cell cytosol. Figures S4–S6: [^1^H, ^15^N] HSQC spectrum of ^15^**N-2** before and after 2 h reduction in cell cytosol. Figures S7–S9: [^1^H, ^15^N] HSQC spectrum of ^15^**N-3** before and after 2 h reduction in cell cytosol. Figure S10: accumulation ratio in A2780 cells treated for 1 h with **1**–**3** in the absence or in the presence of cimetidine. Figure S11: RP-HPLC chromatograms of **3** aged in complete RPMI 1640 at time zero and after 72 h in solution. Figure S12: RP-HPLC chromatograms of **2** aged in RPMI 1640 at time zero and after 72 h in solution, and ESI-MS spectra of **2** and [**2**-Cl + H_2_O]^+^.
