# Supplementary material for: The Strange Case: The Unsymmetric Cisplatin-Based Pt(IV) Prodrug [Pt(CH3COO)Cl2(NH3)2(OH)] Exhibits Higher Cytotoxic Activity with respect to Its Symmetric Congeners due to Carrier-Mediated Cellular Uptake
Source: Bioinorg Chem Appl. 2022 Dec 30;2022:3698391. doi: 10.1155/2022/3698391 (PMC9822769; doi:10.1155/2022/3698391)
Supplement: Supplementary Materials — Figure S1: sketch of the complexes under investigation. Figures S2–S3: [1H, 15N] HSQC spectrum of 15N-1 before and after 2 h reduction in cell cytosol. Figures S4–S6: [1H, 15N] HSQC spectrum of 15N-2 before and after 2 h reduction in cell cytosol. Figures S7–S9: [1H, 15N] HSQC spectrum of 15N-3 before and after 2 h reduction in cell cytosol. Figure S10: accumulation ratio in A2780 cells treated for 1 h with 1–3 in the absence or in the presence of cimetidine. Figure S11: RP-HPLC chromatograms of 3 aged in complete RPMI 1640 at time zero and after 72 h in solution. Figure S12: RP-HPLC chromatograms of 2 aged in RPMI 1640 at time zero and after 72 h in solution, and ESI-MS spectra of 2 and [2-Cl + H2O]+. [file 3698391.f1.pdf]

***The strange case*: the unsymmetric cisplatin-based Pt(IV) prodrug [Pt(CH_3COO)Cl_2(NH_3)$_2$(OH)] exhibits higher cytotoxic activity with respect to its symmetric congeners due to carrier-mediated cellular uptake**

Elisabetta Gabano,[1] Ilaria Zanellato,[2] Giulia Pinton,[3] Laura Moro,[3] Mauro Ravera,[2] and Domenico Osella,[2]

[1] *Dipartimento per lo Sviluppo Sostenibile e la Transizione Ecologica, Università del Piemonte Orientale, Piazza Sant'Eusebio 5, 13100 Vercelli (Italy)*

[2] *Dipartimento di Scienze e Innovazione Tecnologica, Università del Piemonte Orientale, Viale Michel 11, 15121 Alessandria (Italy)*

[3] *Dipartimento di Scienze del Farmaco, Università del Piemonte Orientale, Via Bovio 6, 28100 Novara (Italy)*

Correspondence should be addressed to Mauro Ravera; mauro.ravera@uniupo.it and Domenico Osella; domenico.osella@uniupo.it

SUPPLEMENTARY MATERIAL

Figure S1 Sketch of the complexes under investigation.

Figure S2 [^{1}H, ^{15}N] HSQC spectrum of ^{15}**N-1**.

Figure S3 [^{1}H, ^{15}N] HSQC spectrum of ^{15}**N-1** after 2 h reduction in cell cytosol.

Figure S4 [^{1}H, ^{15}N] HSQC spectrum of ^{15}**N-2**.

Figure S5 [^{1}H, ^{15}N] HSQC spectrum of ^{15}**N-2** after 2 h reduction in cell cytosol.

Figure S6 [^{1}H, ^{15}N] HSQC spectrum of ^{15}**N-2** after 2 h reduction in cell cytosol (magnification).

Figure S7 [^{1}H, ^{15}N] HSQC spectrum of ^{15}**N-3**.

Figure S8 [^{1}H, ^{15}N] HSQC spectrum of ^{15}**N-3** after 2 h reduction in cell cytosol.

Figure S9 [^{1}H, ^{15}N] HSQC spectrum of ^{15}**N-3** after 2 h reduction in cell cytosol (magnification).

Figure S10 Accumulation ratio (AR) in A2780 cells treated for 1 h with **1**-**3** in the absence or in the presence of cimetidine.

Figure S11 RP-HPLC chromatograms of **3** (100 µM) aged in complete RPMI 1640 at time zero and after 72 h in solution.

Figure S12 (A) RP-HPLC chromatograms of **2** aged in RPMI 1640 at time zero and after 72 h in solution. (B) ESI-MS spectra of **2** and [**2**-Cl+H_2O]$^+$. (C) Zoomed-in version of (A).

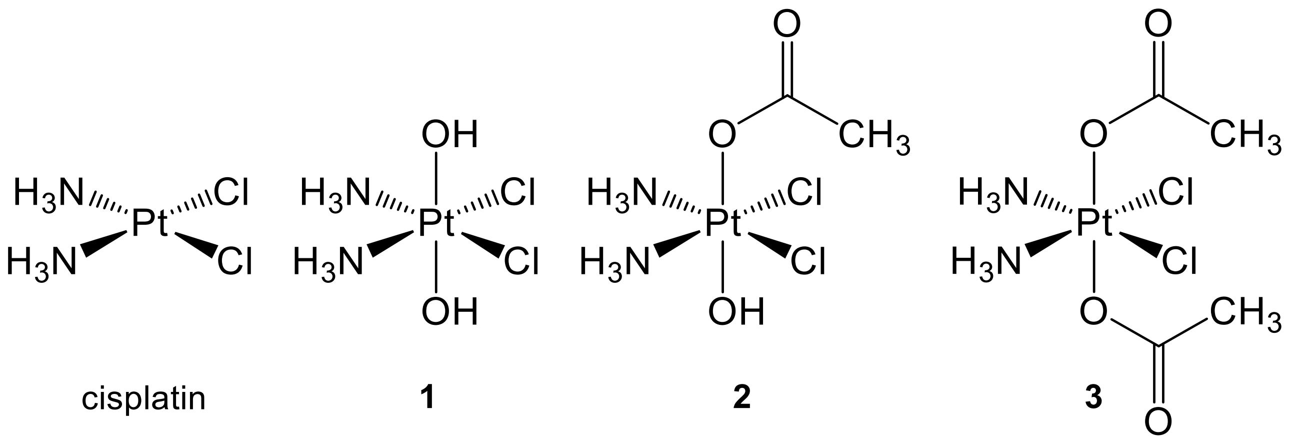

Figure S1 Sketch of the complexes under investigation.

Synthesis of the $^{15}NH_3$-labeled complexes ^{15}N-1, ^{15}N-2, and ^{15}N-3.

The syntheses of ^{15}N-labeled complexes started with the preparation of *cis*-$[PtCl_2(^{15}NH_3)_2]$ as previously reported. ^{15}N-cisplatin was then oxidised with hydrogen peroxide in water to get the dihydroxido complex **^{15}N-1** (with a microwave-assisted heating) or in acetic acid to get the monoacetato complex **^{15}N-2**. Finally, the diacetato complex **^{15}N-3** was obtained from the reaction between **^{15}N-1** and acetic anhydride.

^{15}N-1. Yield: 90 %. ^{15}N NMR (50.70 MHz, H_2O/D_2O 9/1): δ –33.8 ppm with satellite peaks at –31.2 ppm and –36.6 ppm ($^1J_{Pt\text{-}N}$ = 274 Hz and $^2J_{Pt\text{-}H}$ = 52 Hz). ESI-MS (positive ion mode): 336 *m/z* $[M+H]^+$. Calc. for $[Cl_2H_9{}^{15}N_2O_2Pt]^+$ 336 *m/z* $[M+H]^+$.

^{15}N-2. Yield: 85 %. ^{15}N NMR (50.70 MHz, H_2O/D_2O 9/1): δ –35.6 ppm with satellite peaks at –32.9 ppm and –38.2 ppm ($^1J_{Pt\text{-}N}$ = 273 Hz and $^2J_{Pt\text{-}H}$ = 54 Hz). ESI-MS (positive ion mode): 379 *m/z* $[M+H]^+$. Calc. for $[C_2H_{11}Cl_2{}^{15}N_2O_3Pt]^+$ 379 *m/z* $[M+H]^+$.

^{15}N-3. Yield: 80 %. ^{15}N NMR (50.70 MHz, H_2O/D_2O 9/1): δ –39.6 ppm with satellite peaks at –37.0 ppm and –42.5 ppm ($^1J_{Pt\text{-}N}$ = 265 Hz and $^2J_{Pt\text{-}H}$ = 54 Hz). ESI-MS (negative ion mode): 418 *m/z* $[M-H]^-$. Calc. for $[C_4H_{11}Cl_2{}^{15}N_2O_4Pt]^-$ 418 *m/z* $[M-H]^-$.

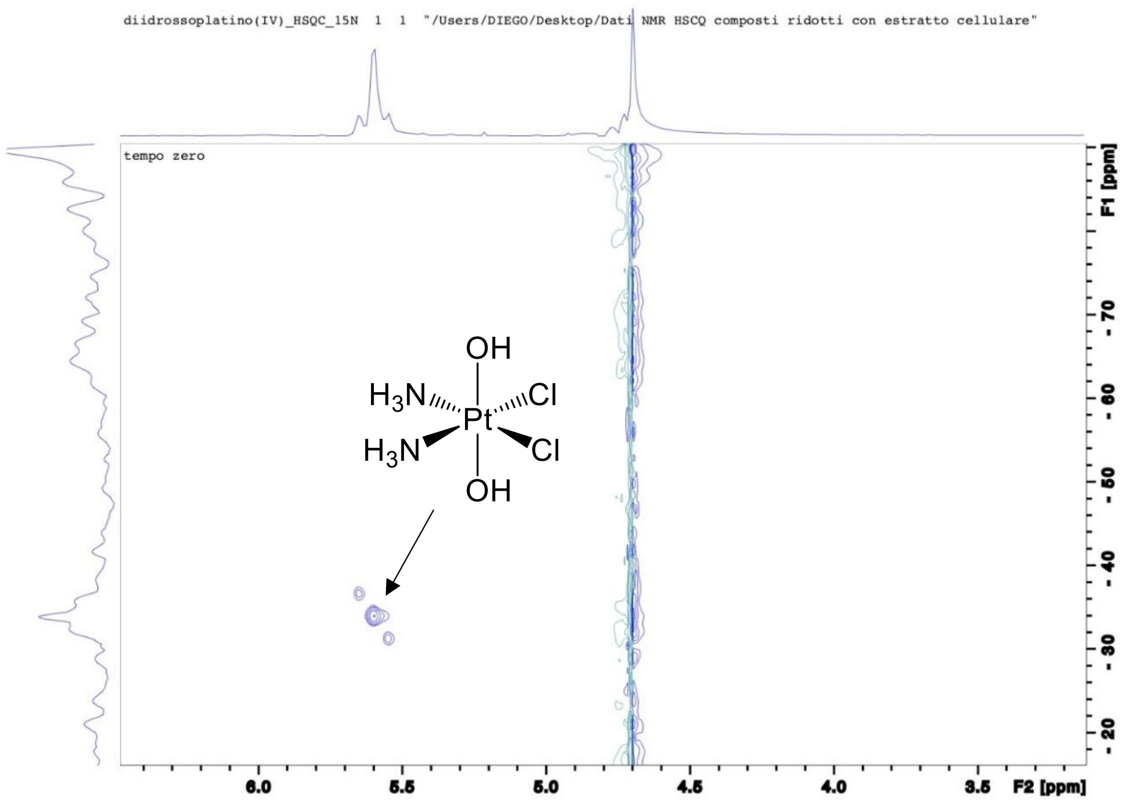

Figure S2 [^{1}H, ^{15}N] HSQC spectrum of **^{15}N-1** in H_2O/D_2O 9/1.

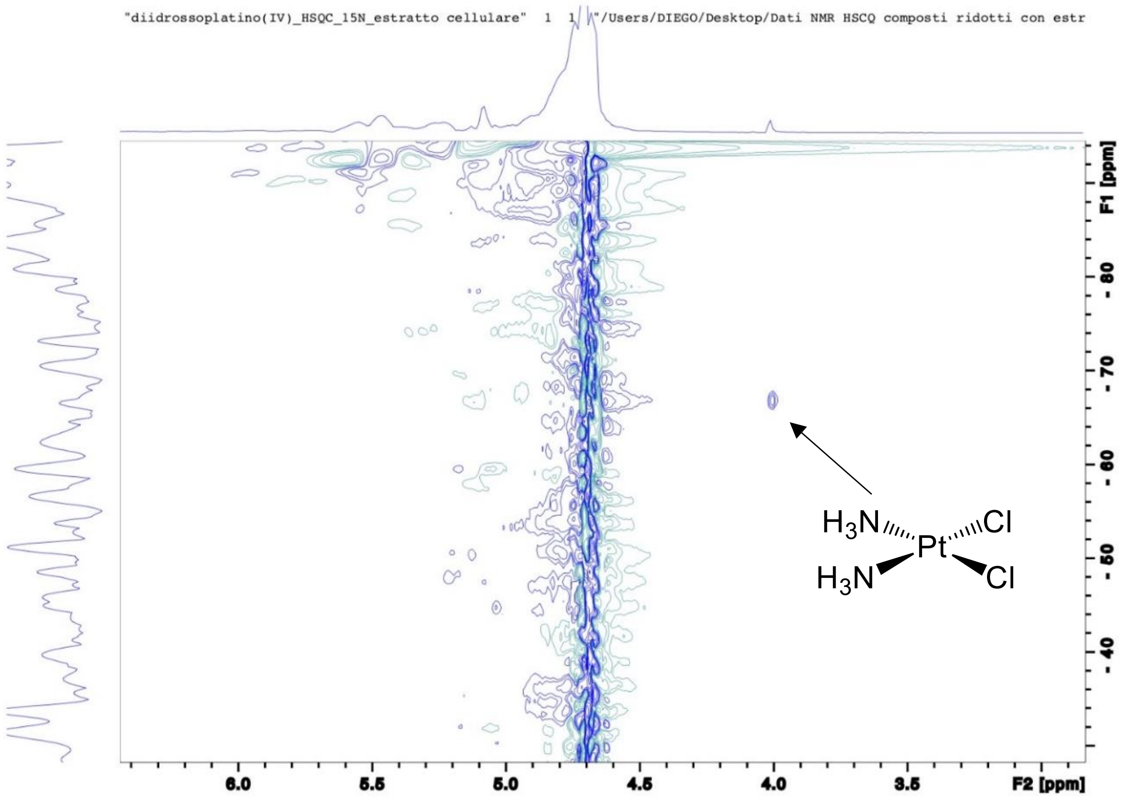

Figure S3 [^{1}H, ^{15}N] HSQC spectrum of **^{15}N-1** after 2 h reduction in cell cytosol.

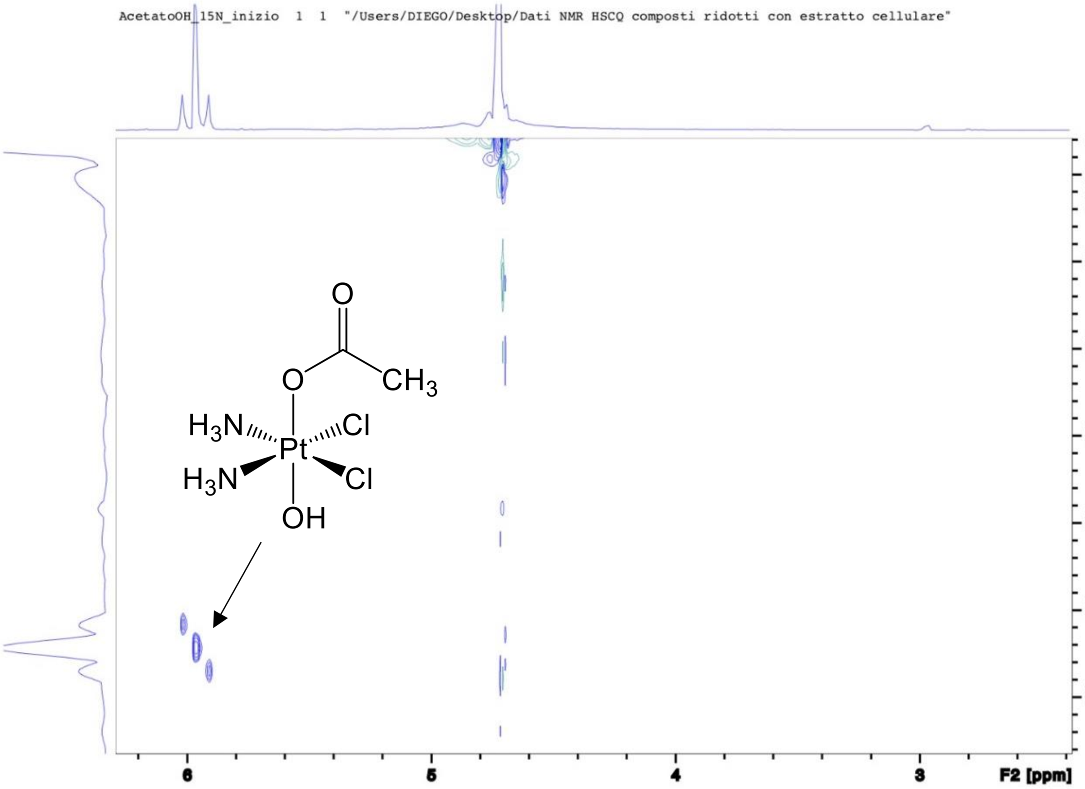

Figure S4 [^{1}H, ^{15}N] HSQC spectrum of **^{15}N-2** in H_2O/D_2O 9/1.

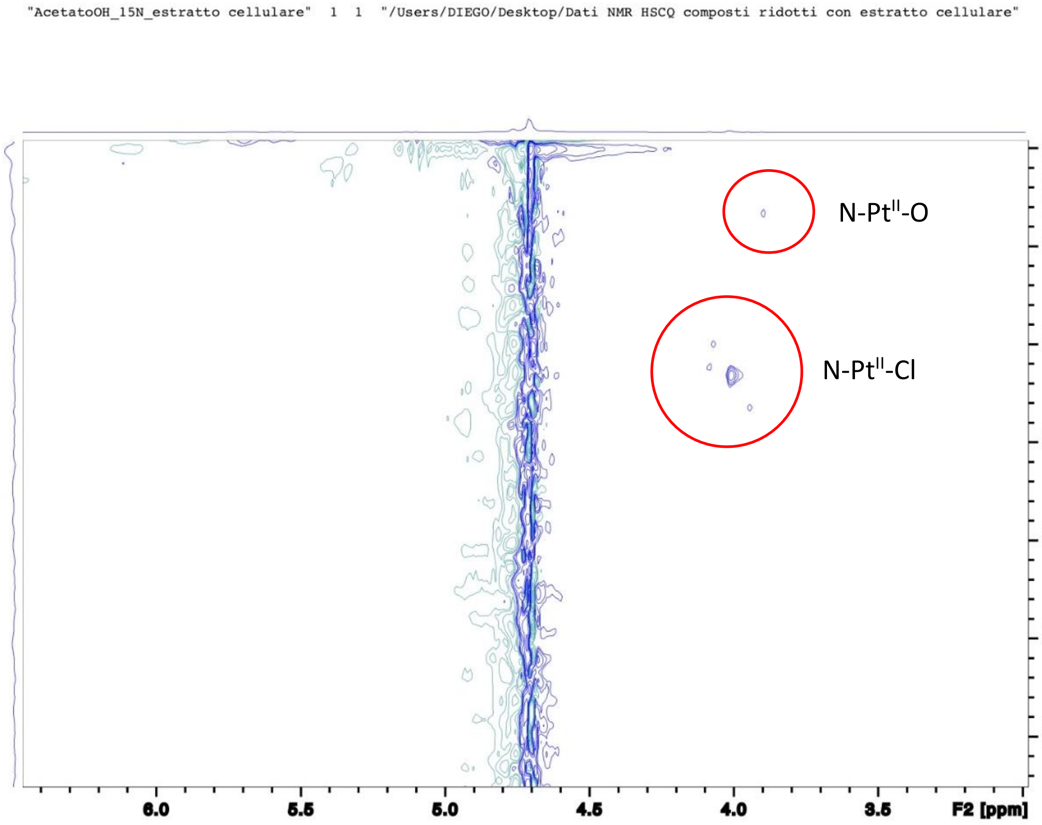

Figure S5 [^{1}H, ^{15}N] HSQC spectrum of **^{15}N-2** after 2 h reduction in cell cytosol.

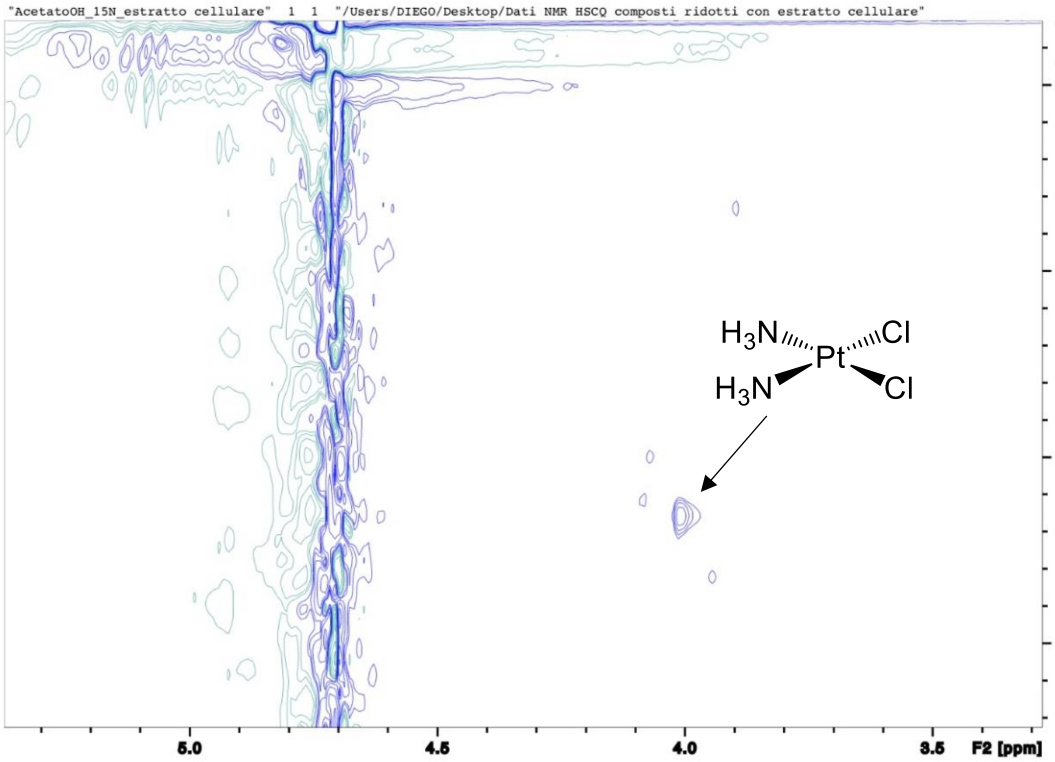

Figure S6 [^{1}H, ^{15}N] HSQC spectrum of ^{15}**N-2** after 2 h reduction in cell cytosol (magnification).

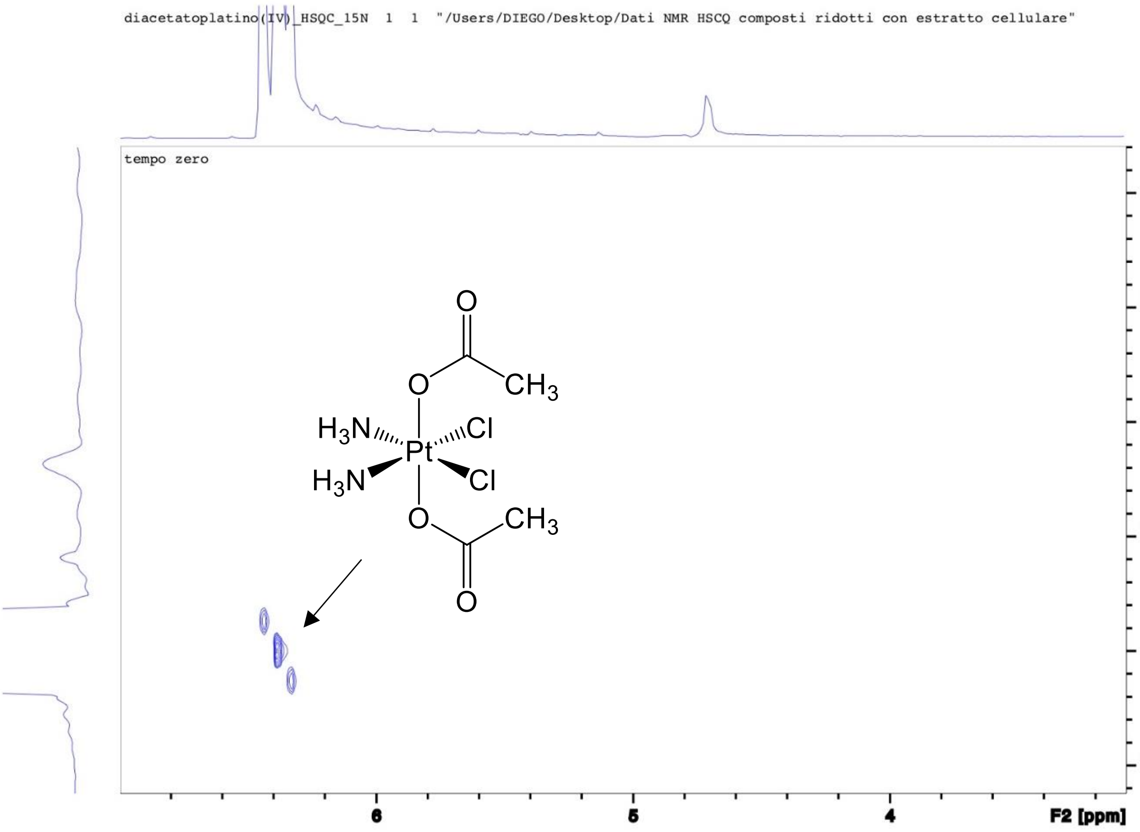

Figure S7 [^{1}H, ^{15}N] HSQC spectrum of ^{15}**N-3** in H_2O/D_2O 9/1.

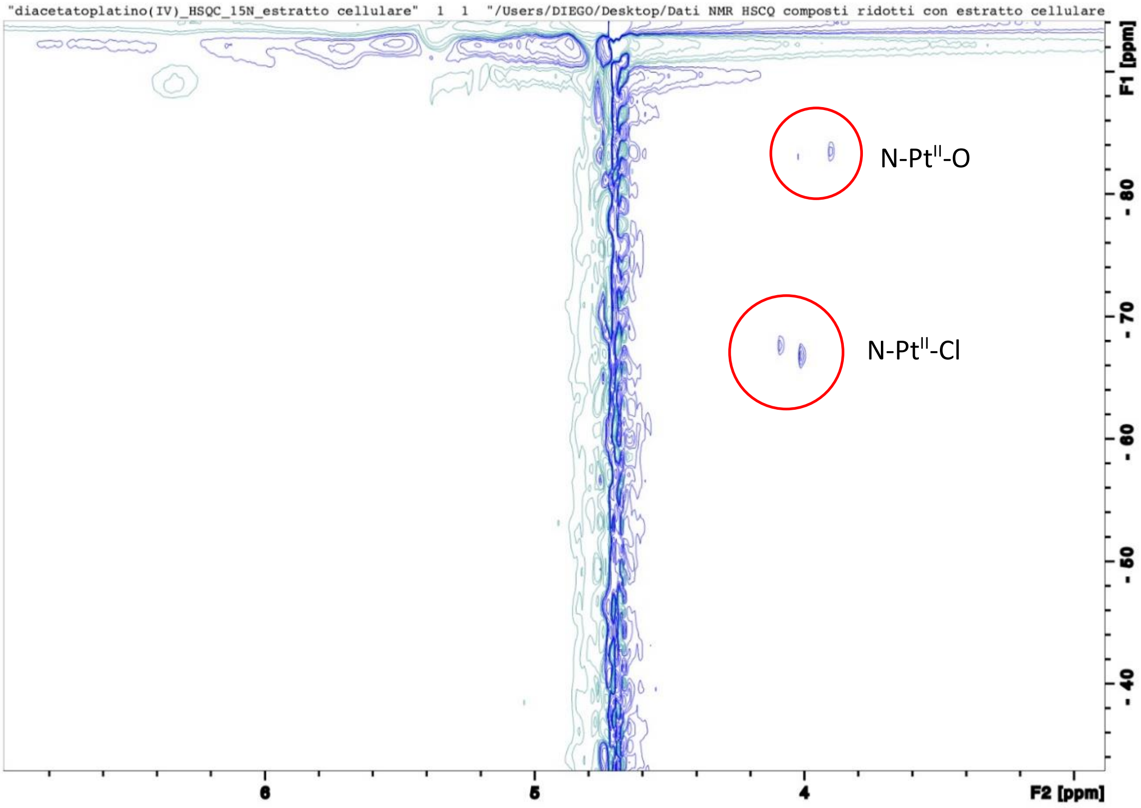

Figure S8 [^{1}H, ^{15}N] HSQC spectrum of **^{15}N-3** after 2 h reduction in cell cytosol.

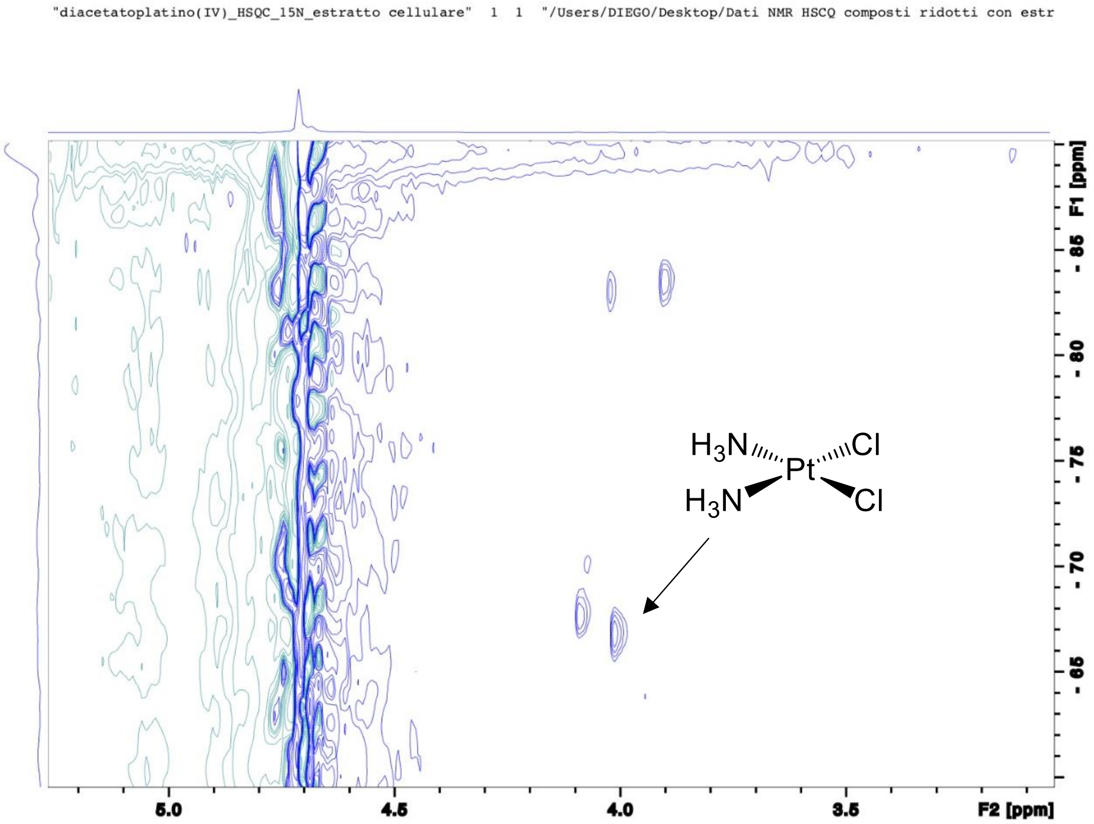

Figure S9 [^{1}H, ^{15}N] HSQC spectrum of **^{15}N-3** after 2 h reduction in cell cytosol (magnification).

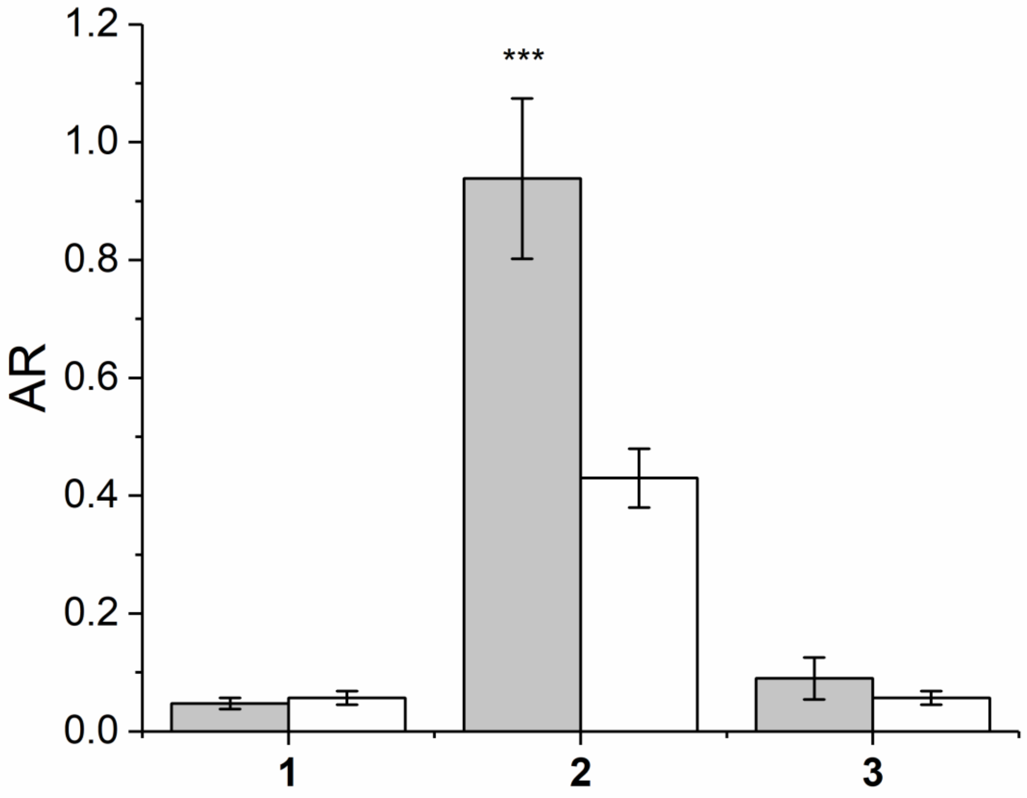

Figure S10 Accumulation ratio (AR) in A2780 cells treated for 1 h with **1**-**3** (10 µM) in the absence (gray bars) or in the presence (white bars) of cimetidine (CMT, co-treatment, 1.5 mM). Data are the mean ± sd of three independent replicates and were compared using one-way analysis of variance (ANOVA) with Tukey post-hoc test. Statistical analysis (in presence *vs.* in absence of CMT): no indication = not significant; ***$p < 0.001$).

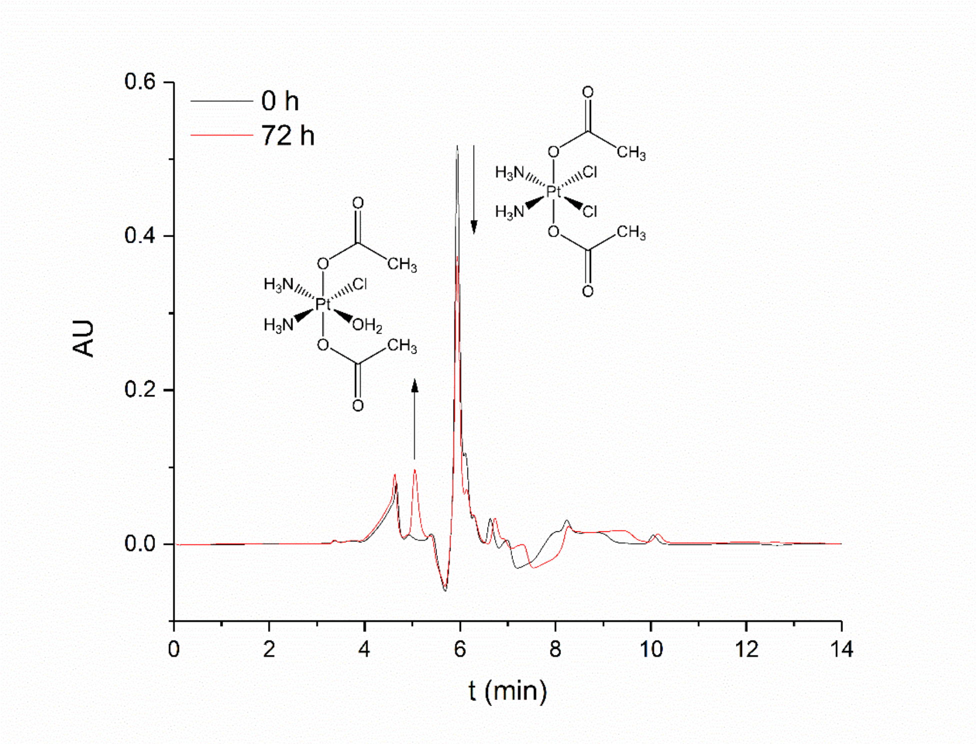

Figure S11 RP-HPLC chromatograms of **3** (100 µM) aged in complete RPMI 1640 medium at time zero and after 72 h in solution.

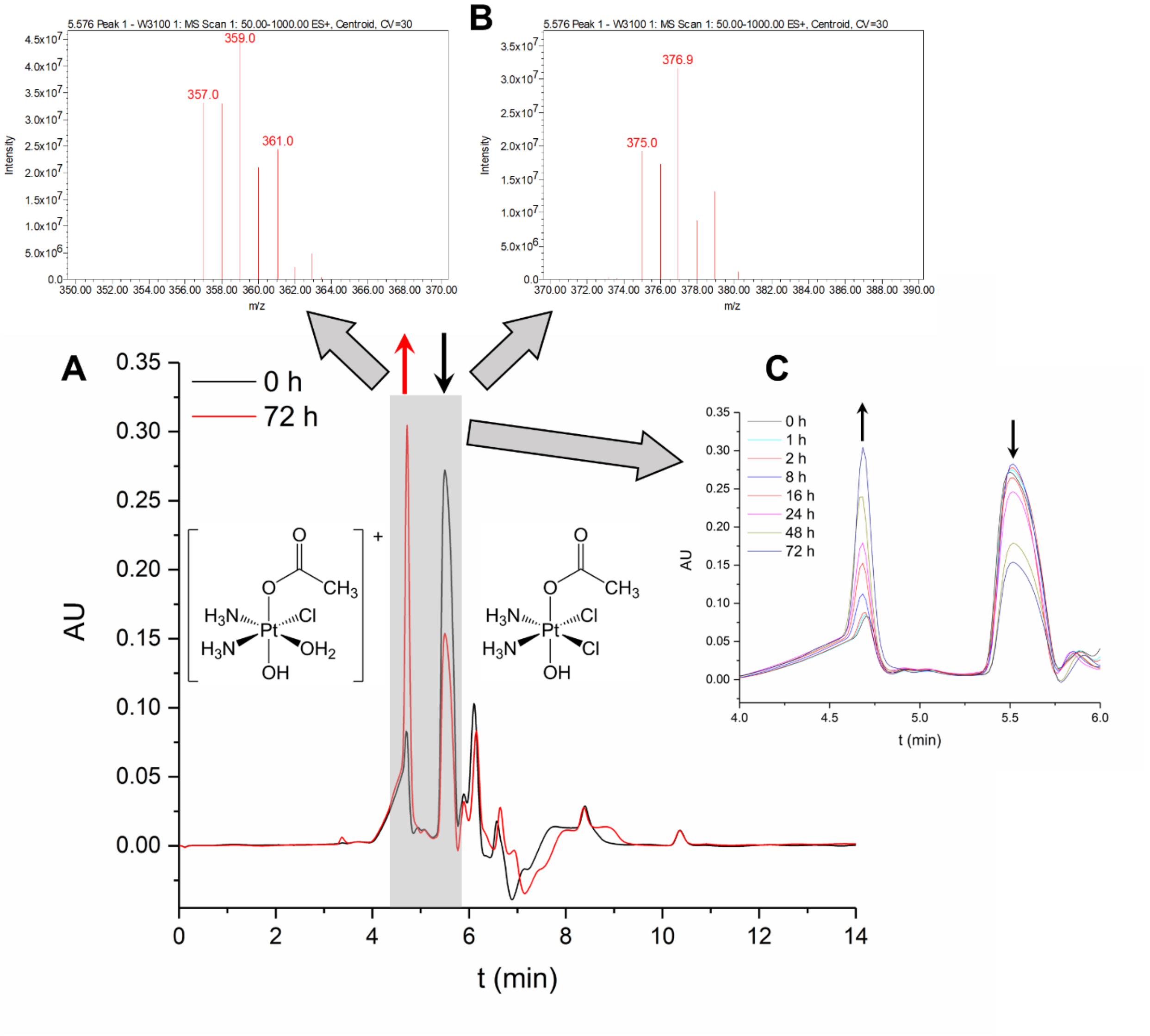

Figure S12 (**A**) RP-HPLC chromatograms of **2** (100 µM) aged in complete RPMI 1640 medium at time zero and after 72 h in solution. In the gray box, the two peaks belonging to **2** and its hydrolyzed derivative [**2**-Cl+H_2O]$^+$ are present. (**B**) ESI-MS spectra of **2** and [**2**-Cl+H_2O]$^+$. (**C**) Zoomed-in version of **A** in the gray box area containing the chromatograms at t = 0 and 72 h, and those obtained at intermediate aging times. (Note: the peaks below 0.10 AU intensity belong to the medium, and partially overlap the peaks of interest, but the hyphenated technique permits identification of the Pt species).
